# Prevalence and predicors of COVID-centred obsessive compulsive disorder among Iranian COVID-19 recovered individuals: a Bayesian analysis

**DOI:** 10.1186/s12888-023-04762-4

**Published:** 2023-05-03

**Authors:** Amir Hossein Shafighi, Foroozan Atashzadeh-Shoorideh, Abbas Ebadi, Fataneh Ghadirian

**Affiliations:** 1 Student Research Committee, School of Nursing and Midwifery, Shahid Beheshti University of Medical Sciences, Tehran, Iran; 2 Psychiatric Nursing and Management Department, School of Nursing and Midwifery, Shahid Beheshti University of Medical Sciences, Tehran, Iran; 3 Behavioral Sciences Research Center, Nursing Faculty, Baqiyatallah University of Medical Sciences, Tehran, Iran

**Keywords:** Obsessive compulsive disorder, COVID-19, Recovery, Mental health

## Abstract

**Background:**

The evidence on the psychological consequences of coronavirus 2019 mainly relates to general psychiatric problems, and a few studies have reported the incidence and predictors of obsessive-compulsive disorder.

**Objective:**

To determine the prevalence of obsessive-compulsive disorder (OCD) and its predictors in Iranian COVID − 19 recovered individuals at 3–6 months, 6–12 months, and 12–18 months after recovery.

**Method:**

In this cross-sectional analytical study, 300 participants were randomly selected based on the inclusion criteria from three hospitals in three different regions of Tehran, Iran, and were assessed by the Clinical Demographic Information Questionnaire, the Obsessive Compulsive Inventory-Revised (OCI-R), the Depression, Anxiety and Stress Scale 21 (DASS21), The Pittsburgh Sleep Quality Index (PSQI) and Posttraumatic Stress Disorder Checklist for DSM-5 (PCL-5). The obtained data were analyzed with SPSS version 26.

**Results:**

The results showed that the mean score of OCD is 30.58 ± 15.22, with a prevalence of 71% (n = 213). Female gender (BF = 0.50, p = 0.01), sleep disturbance (BF = 0.02, p = 0.001), PTSD (BF = 0.009, p = 0.0001), depression (BF = 0.0001, p = 0.0001), and stress (BF = 0.0001, p = 0.001) are the strongest predictors of the presence of OCD in recovered COVID − 19 individuals.

**Conclusion:**

OCD-like symptoms was observed in the majority of COVID − 19 recovered individuals with mild to moderate severity. In addition, the stated prevalence, severity, and significance varied according to sociodemographic and health inequalities.

## Background

Although most COVID − 19 infected individuals have recovered from the acute sequelae such as Acute Respiratory Distress Syndrome (ARDS), they still suffer from some of the remaining chronic sequelae. As a result, some of them had a new or even worsened health condition after discharge from the hospital because of their experience with ARDS and mechanical ventilation [1, 2]. In this regard, the psychological sequels of COVID-19 are considerable [3, 4]. Many studies have indicated the manifestations of common psychological problems among COVID-19 recovered individuals even eighteen months following its onset [5, 6]. Disorders such as anxiety, depression, sleep disturbances, and obsessive behaviors have been observed in COVID-19 recovered individuals [7, 8]. The psychological problems may be secondary to the disease, the therapeutic process, or the stress during the pandemic [9]. In contrast, some studies came to different conclusions and considered that the majority of COVID − 19 recovered individuals were able to approximately regain their previous psychological and social well-being after recovery from the disease [10].

While there are fewer reports, research shows that the prevalence of Obsessive-Compulsive Disorder (OCD) symptoms increased during the COVID-19 pandemic at a significantly higher rate than pre-pandemic rates [11]. Owji et al. (2022) presented obsessive-compulsive disorder as a manifestation of COVID − 19 in an Iranian case series [12]. OCD is shown to be associated with low-grade inflammation, neuroinflammatory changes in the brain, and microglial activation after COVID-19 infection as potential etiologic factors [11].

OCD as a chronic and life disrupting psychological tendency, characterized by manifestations of excessive repetitive obsessive thoughts and irresistible compulsive behaviors [13]. OCD is prevalent among 2–3% of the worldwide population. Though the majority of individuals with OCD respond to therapeutic measures as pharmacotherapy and Cognitive Behavioral Therapy (CBT), still some individuals recuperate only with surgical implementations, such as anterior capsulotomy and cingulotomy [14].

As claimed by studies, regardless of the fact that OCD is an indisputable psychological disorder, it is meaningfully correlated with other psychological disorders such as anxiety disorders [15, 16]. Hence, the COVID-19, as an anxiety-related factor, is affiliated with OCD either directly or indirectly [17]. In addition, certain compulsive behaviors may be complicated by factors such as the increased need for hand washing, which is considered one of the safest precautions for preventing infection COVID − 19 [18].

Some studies examining COVID-centered Obssesive-Compulsive Symptoms estimated the pool incidence of new, COVID-centred obsessions and compulsions at 55% in the general population and 16% in the clinic [19]. Recent studies assessing psychiatric symptoms in COVID − 19 survivors 1 month after infection found that approximately 20% reported obsessive-compulsive symptoms [11]. However, obsessive-compulsive symptoms after COVID-19 infection are rarely reported in the literature.

Indeed, the psychiatric manifestations of COVID − 19 are a biopsychosocial phenomenon. Therefore, the causes and roots of the emerging obsessive-compulsive disorder in survivors of COVID − 19 are affected by psychosocial differences in addition to biological mechanisms.

Simultaneously, the inequality in socio-demographic status, the level of social welfare and health services accessibility is one of the substantial challenges determining the overall health of individuals [20, 21]. In this regard, based on studies, the stated inequalities have exhaustively influenced the course of COVID-19 in the form of pandemic supervisory, therapeutic and vaccination affairs [22, 23].

Despite the fact that studies in this realm have been inadequate and concluded to incoherent outcomes, consequences such as pathological obsessive behaviors following the psychological impairments of COVID-19, even after recovery and discharge from the hospital, emphasized the noteworthiness of study in this specific area [24].

Moreover, the fact that mentioned impairments generally remain unrecognized until they progress to severe functional disturbances, emphasize the importance of providing psychological screening for COVID-19 recovered individuals [3, 4]. As called for in Lancet, community mental health services need to be established to screen COVID − 19 recovered individuals [25]. The devastating psychological sequels of COVID-19, particularly the OCD, alongside the durability of mentioned sequels even long after recovery, underline the significance of investigating the mental health status of COVID-19 recovered individuals aiming to broaden the structured community-based interventions. Concurrently, the stated correlation and predictive power between socio-demographic inequalities and the prevalence of psychological consequences of COVID-19, amplify the necessity of implementation of present study. Therefore, the present study was conducted in order to look at the prevalence of new-onset OCD in Iranian COVID-19 survivors and its socio-demographic and health predictors at 3–6 months, 6–12 months, and 12–18 months after recovery.

## Methods

### Study design and sites

The present study was a cross-sectional analytic study conducted between May 2021 and July 2022 to determine the prevalence of OCD and sociodemographic and health disparities among COVID − 19 recovered individuals.

Based on the geographical distribution of the study areas, three hospitals from three regions in the south, center, and north of Tehran were randomly selected by lottery. The selected hospitals were Shahid Taleghani Hospital in the north (Hospital A), Imam Hossein Hospital in the center (Hospital B), and finally Loghman Hakim Hospital in the south of Tehran (Hospital C). It should be noted that there is a socio-economic divide between the northern, central and southern regions of Tehran. The residents of the northern region of Tehran are considered to have a higher socioeconomic status. In contrast, the residents of the southern region are attributed the lowest socioeconomic status, and finally, the residents of the central region belong to the middle socioeconomic status. After randomly selecting the samples in each hospital using a table of random numbers, the selected samples were matched with the inclusion and exclusion criteria. The above method was continued continuously until the expected sample size was reached in each hospital (n = 100).

### Population and data collection

The study population consisted of COVID − 19 recovered individuals discharged from either Shahid Beheshti University of Medical Sciences hospitals or hospital emergency departments after the initial examination. Sample size was calculated based on the first sample (pilot study) and the lowest probability of occurrence of psychocognitive disorders (effect size: 1.4) using G-Power version 3.1 software. The significance level was set at p < 0.05.

The samples initially consisted of individuals infected with COVID − 19 with moderate, severe, and critical severity and in whom at least 12 weeks (3 months) and no more than 60 weeks (18 months) had elapsed since the onset of acute symptoms of COVID − 19. The initial sample, obtained from individuals’ medical records, included 11,337 individuals, of whom 2117 were randomly selected based on the study’s inclusion and exclusion criteria. After information was provided, 1103 individuals provided informed consent while meeting sufficient criteria to participate in the study. Ultimately, 339 individuals completed the entire questionnaire. To ensure geographic equality, 100 individuals were randomly selected for each hospital, resulting in a total number of 300 individuals.

Inclusion criteria included participant age between 18 and 65 years; the presence of a COVID − 19 diagnosis based on diagnostic criteria and records registered in hospitals or treatment centers; disease severity in the moderate, severe, or critical range (according to Table 1); the elapse of at least 12 weeks (3 months) and no more than 60 weeks (18 months) since the onset of symptoms; the need for voluntary participation by participants, the indispensability of sufficient knowledge and decision-making skills of the participants, the absence of current or previous psychiatric disorders of the participants, the absence of current or previous substance abuse of the participants, the absence of severe or disabling physical impairments of the participants, and finally, the absence of a psychological period of grief or loss due to the death or loss of a loved one. On the other hand, exclusion criteria were that more than 10% of the questionnaires remained uncompleted and that participants tended to drop out of the study.

**Table 1:** Coronavirus 2019 classification based on signs and symptoms severity

Severity	Signs and Symptoms
Asymptomatic	♣ Nucleic acid test for coronavirus 2019 is positive. ♣ Absence of clinical signs and symptoms. ♣ Normal chest imaging tests
Mild	♣ Upper respiratory tract infection (fever, fatigue, myalgia, cough, sore throat, runny nose, sneezing) ♣ Gastrointestinal symptoms (nausea, vomiting, abdominal pain or diarrhea).
Moderate	♣ Pneumonia (recurrent fever and cough) without hypoxemia. ♣ The chest imaging tests are indicating the lesions.
Severe	♣ Pneumonia with hypoxemia (Spo2 < 92%)
Critical	♣ Acute Respiratory Distress Syndrome, Encephalopathy, Myocardial Injury, Heart Failure, Coagulation Disorders and Acute Kidney Injury

### Measures of variables

The research instruments used in the present study were the “Clinical Demographic Information Questionnaire” and the “Obsessive Compulsive Inventory - Revised (OCI-R).“ The “Clinical Demographic Information Questionnaire,“ which was created and edited by the authors, included questions on age, sex, marital status, educational level, employment status, economic status, number of offspring, place of birth, place of residence, type of residence, health status, previous illnesses, Medication history, minimum arterial oxygen saturation (SaO2) during hospitalization, number of days spent in hospital due to COVID − 19, number of days recovered from COVID − 19, and finally history of hospitalization in intensive care unit (ICU). To assess the validity of the “Clinical Demographic Information Questionnaire,“ the qualitative content validity method was used.

Another applied tool was the Persian version of the OCI-R. The OCI-R was first introduced in 2002 by Foa et al. with the aim of assessing obsessive-compulsive disorder. This instrument included the components “Washing,“ “Obsessing,“ “Neutralising,“ “Ordering,“ “Hoarding,“ and finally “Checking” each of which consisted of 3 items. The OCI-R included a total of 18 items, which were rated on a five-point Likert scale ranging from 0 to 4. Thus, the total score of this instrument ranged from 0 to 72, and a score of 21 or more indicated the presence of OCD.

To determine the validity of this questionnaire, a comparison was made between this instrument and the “Maudsley Obsessive-Compulsive Inventory (MOCI),“ in which the Pearson correlation coefficient for all scales and subscales was 0.61 to 0.75. To determine the reliability of this instrument, the test-retest method was used over a 2-week period, and the result ranged from 0.77 to 0.97 [26]. The Chronbach alpha of the OCI-R was determined to be 0.76 in the present study.

To determine the correlation and predictive power of the variables “stress,“ “anxiety,“ “depression,“ “sleep disturbance,“ and “PTSD,“ the Persian versions of the Depression, Anxiety and Stress Scale 21 (DASS21), the Pittsburgh Sleep Quality Index (PSQI), and the Posttraumatic Stress Disorder Checklist for DSM-5 (PCL-5) were used. The validity and reliability of the above instruments were acceptable [27–29]. The reliability values (according to Chronbach Alpha) of all these scales examined in the present study ranged from 0.65 to 0.87.

After approval of the proposal by the Ethics Committee of Shahid Beheshti College of Medical Sciences, the Code of Ethics was obtained. Subsequently, the necessary approvals for access to the research sites were obtained. Sampling in the selected hospitals was based on the medical records of the participants using a table of random numbers. They were then matched with the inclusion and exclusion criteria of the study. The final sample was then determined and participants were contacted by telephone. They were then provided with additional information about the study and their rights. Then, the informed consent form was sent electronically to each participant via WhatsApp and their electronic signature was obtained. In the present study, the data collection instruments were used in the form of self-report questionnaires, which were created as an electronic online link in the “Porsa_Irandoc” system. Then, the links were sent to each person via WhatsApp along with additional information. The questionnaires were also designed to be easy to read, and each question was displayed on a separate page.

### Statistical analysis

Finally, after the data were collected, they were analyzed using descriptive and inferential statistics according to the nature of the variables and the purpose of the study using SPSS version 26, taking into account the level of test error and the significance level of 0.05. The frequency of the quantitative variables of the participants was expressed as mean and statistical standard deviation, while the qualitative variables were expressed as relative frequency. The analysis of the relationships between the sociodemographic and health inequalities and the subscales of OCD was indicated by the statistical values ANOVA and chi-square. Finally, Bayesian linear regression test was used to evaluate the correlation and predictive power between OCD and sociodemographic and health inequalities.

### Ethical considerations

Considering that adherence to ethical principles in the conduct of any research study is uncontroversial, this study was approved by the Shahid Beheshti Medical University (SBMU) Ethics Committee (IR. SBMU. PHARMACY. REC.1400.068). In addition, approval was also obtained from the Vice Chancellor for Research Affairs at SBMU. According to the ethical codes and the rights of the participants, the participants were allowed to leave the study whenever they wanted. The researcher was required to keep the participants’ information confidential and was not allowed to disclose it, and if disclosure of information was necessary due to the circumstances, the researcher was required to inform the participants before disclosure; participants were also given the first author’s phone number so that they could report any complications or problems related to the study; ethical principles in the use of other study sources were considered; fidelity and accuracy in sampling, data collection, and data analysis were considered; and finally, the structures and methods used in the present study did not conflict with the religious and cultural norms of the participants and society.

## Results

### Socio-demographic and clinical characteristics

A total of 300 subjects participated in this study, including 100 subjects for hospital A in the north of Tehran (33.3%), 100 subjects for hospital B in the center of Tehran (33.3%), and finally 100 subjects for hospital C in the south of Tehran (33.3%). In addition, most of the participants were female (55.3%), had a higher educational level (59.7%), were employed (53.3%), married (88%), and were hospitalized (79.3%). In addition, only 34 participants (11.3%) and 24 participants (8%) of the total 300 participants were admitted to the ICU or had an intubation history, respectively (Table 2). The age of the participants ranged from 18 to 65 years, with a mean age of 41.69 ± 9.06 years. In addition, the mean number of days of hospitalization of the participants was 5.33 ± 3.61. From the records, the mean value of minimum arterial oxygen saturation (SaO2) in participants at the time of initial diagnosis of COVID − 19 was 78.99%, with a standard deviation of 9.99.

**Table 2:** Sociodemographic and medical characteristics of recovered COVID-19 patients

	N	Hospital A	Hospital B	Hospital C
	Frequency(%)	Frequency(%)	Frequency(%)	Frequency(%)
*Gender*				
Male	134 (44.7)	53 (53.0)	36 (36.0)	45 (45.0)
Female	166 (55.3)	47 (47.0)	64 (64.0)	55 (55.0)
*Educational level*				
Primary	66 (22.0)	21 (21.0)	17 (17.0)	28 (28.0)
Secondary	55 (18.3)	21 (21.0)	13 (13.0)	21 (21.0)
Higher	179 (59.7)	58 (58.0)	70 (70.0)	51 (51.0)
*Occupational status*				
Unemployed	124 (41.4)	65 (65.0)	44 (44.0)	51 (51.0)
Employed	160 (53.3)	32 (32.0)	49 (49.0)	43 (43.0)
Retired	16 (5.3)	3 (3.0)	7 (7.0)	6 (6.0)
*Marital status*				
Unmarried	36 (12.0)	9 (9.0)	13 (13.0)	14 (14.0)
Married	264 (88.0)	91 (91.0)	87 (87.0)	86 (86.0)
*Hospitalization*				
Yes	238 (79.3)	75 (75.0)	83 (83.0)	80 (80.0)
No/Referred to home	62 (20.7)	25 (25.0)	17 (17.0)	20 (20.0)
*Admission to ICU*				
Yes	34 (11.3)	13 (13.0)	10 (10.0)	11 (11.0)
No	266 (88.7)	87 (87.0)	90 (90.0)	89 (89.0)
*Intubation*				
Yes	24 (8.0)	13 (13.0)	4 (4.0)	7 (7.0)
No	276 (92.0)	87 (87.0)	96 (96.0)	93 (93.0)
*Time elapsed since onset of symptoms*				
3 to 6 months	100 (33.3)	36 (36.0)	17 (17.0)	47 (47.0)
6 months to 1 year	100 (33.3)	31 (31.0)	22 (22.0)	47 (47.0)
1 year to 1.5 years	100 (33.3)	33 (33.0)	61 (61.0)	6 (6.0)

### Prevalence and severity of OCD

In this study, the prevalence and severity of OCD in COVID − 19 recovered individuals were assessed using the OCI-R. According to this instrument, the total score of the OCI-R ranged from 0 to 72, and the mean total score of the OCI-R was 30.58 (SD = 15.22). Consequently, a total score of 21 or more was considered the presence of OCD. Based on the data obtained, 213 (71%) subjects had a total score of 21 or more, indicating the presence of OCD.

### Sociodemographic, health history, and mental health disparities between the subjects with and without OCD

There is a significant difference in minimum SaO2 and number of hospitalization days, gender, place of residence, hospital admitted, and history of ICU admission between subjects with and without OCD (Table 3). The difference in mental health status between subjects with and without OCD is remarkable (Table 4).

**Table 3:** Sociodemographic and health disparities between the subjects with and without OCD

Variables		OCI-R level		Statistics
		No OCD (below 21)	Presence of OCD (21 and above)	
		Mean(SD)	Mean(SD)	t (df), p- value
**Age**		41.45(8.71)	41.79(9.21)	t=-0.296 (298), p = 0.76
**Minimum SaO**_**2**_		81.28(9.23)	78.05(10.16)	t = 2.55 (298), p = 0.01*
**Number of hospitalization days**		4.66(3.18)	5.60(3.74)	t=-2.06 (298), p = 0.03*
		**n (%)**	**n (%)**	**χ2 (df), p- value**
**Gender**	**Male**	48 (35.82%)	86 (64.17%)	χ2 = 5.47 (1), p = 0.01*
	**Female**	39 (23.49%)	127 (76.50%)	
**Place of residence**	**Tehran**	79 (27.43%)	209 (72.56%)	χ2 = 17.92 (7), p = 0.01*
	**Other Cities**	8 (66.66%)	4 (33.33%)	
**Hospital admitted**	**A**	23 (23%)	77 (77%)	χ2 = 10.49 (2), p = 0.005*
	**B**	41 (41%)	59 (59%)	
	**C**	23 (23%)	77 (77%)	
**ICU admission**	**No**	82 (30.82%)	184 (69.17)	χ2 = 3.80 (1), p = 0.05*
	**Yes**	5 (14.70%)	29 (85.29%)	
**Marital status**	**Married**	76 (28.78%)	188 (71.21%)	χ2 = 0.04 (1), p = 0.82
	**Single**	11 (30.55%)	25 (69.44%)	
**Intubation**	**No**	84 (30.43%)	192 (69.56%)	χ2 = 3.44 (1), p = 0.06
	**Yes**	3 (12.50%)	21 (87.50%)	
**Time elapsed from onset**	**3 to 6 month**	31 (31%)	69 (69%)	χ2 = 2.72 (2), p = 0.25
	**6 to 12 month**	23 (23%)	77 (77%)	
	**12 to 18 month**	33 (33%)	67 (67%)	
**Hospitalization**	**No**	71 (29.83%)	167 (70.16%)	χ2 = 0.38 (1), p = 0.53
	**Yes**	16 (25.80%)	46 (74.19%)	

*p < 0.05

**Table 4:** Mental health disparities between the subjects with and without OCD

Variables	OCI-R level		Statistics
	No OCD (below 5)	Presence of OCD (21 and above)	
	Mean (SD)	Mean (SD)	T_df_, p- value
**PTSD**	30.24 (19.21)	40.67 (15.67)	T_298_=-4.88, p = 0.001^**^
**Sleep disorder**	5.24 (3.58)	6.26 (2.92)	T_298_=-2.55, p = 0.01^*^
**Depression**	3.13 (3.19)	10.96 (4.26)	T_298_=-15.43, p = 0.001^**^
**Anxiety**	3.29 (3.38)	10.27 (3.91)	T_298_=-14.54, p = 0.001^**^
**Stress**	3.78 (3.61)	10.98 (3.93)	T_298_=-14.71, p = 0.001^**^

*p < 0.05

**p < 0.01

### Predictors of OCD in COVID-19 recovered individuals

The results show that stress, depression, anxiety, PTSD, sleep disturbance, and female gender are the predictors of OCD in the subjects (p < 0.05) (Table 5). The closer the Bayesian value is to zero, the higher the predictive power is. The results show that the above predictors have high predictive power for OCD as a response condition in COVID − 19 recovered subjects.

**Table 5:** Prediction of OCD among recovered COVID-19 individuals based on linear Bayesian regression

Response variable	Exploratory variables	Bayesian factor	p-value
**OCD**	**Stress**	0.0001	0.001**
	**Depression**	0.0001	0.0001**
	**Anxiety**	0.0001	0.0001**
	**PTSD**	0.009	0.0001**
	**Sleep disorder**	0.02	0.001**
	**Female**	0.50	0.01*
	**Hospital admitted**	1.44	0.05
	**Intubation history**	0.76	0.06
	**ICU Admission history**	0.72	0.05
	**Fewer days of hospitalization**	0.09	0.05

*p < 0.05

**p < 0.01

## Discussion

The present study was conducted with the aim of determining the prevalence of OCD and sociodemographic and health disparities among COVID − 19 recovered individuals in Iran. The results show that OCD was found in more than half (71%) of COVID − 19 recovered individuals in the present study. Because the OCI R is a self-assessment instrument, it should be considered that these patients with a score greater than 21 are likely to have significant OCD-like symptoms and no OCD diagnosis. Uvais (2021) reported that 20% of COVID − 19 survivors had the obsessive-compulsive symptoms 1 month after infection [11].

However, the severity and frequency of the reported disorder varied among participants depending on different variables. However, on average, the severity of OCD was rated as mild to moderate among participants in the present study (mean = 30.58) and none of them was under psychiatric treatment for his obsessive-compulsive symptoms.

Khosravani et al. (2021) examined the Iranian context for comparing OCD before and during the coronavirus pandemic. They discussed that OCD symptoms and severity were strongly related to stress responses associated with the coronavirus [30]. Shabani et al. (2023) considered that the prevailing health anxiety related to uncertainty in Iranian society is a central factor for mental disorders in the period COVID − 19 [31]. Dehghani et al. (2022) noted that two years after the emergence of coronavirus and the reduction of fear and anxiety, the manifestations of obsessive-compulsive disorder in Iranian adults appeared to decrease [32].

Furthermore, in the present study, individuals with OCD were older and, at the same time, had lower minimum SaO2 values and, conversely, more hospitalization days than individuals without OCD. However, the aforementioned differences were significant only for the variables “minimum SaO2 value” and “number of hospitalization days”. As Abba-Aji et al. (2020) stated, there is a direct and significant relationship between the prevalence of OCD after the pandemic COVID − 19 and age. In other words, OCD was more prevalent in older individuals [33]. A study conducted by Sreevalsan et al. in 2020 to determine the impact of length of hospital stay on the extent of vulnerability of COVID − 19 recovered individuals in Singapore found that longer length of hospital stay was associated with higher extent of vulnerability of COVID − 19 recovered individuals [34].

In addition, the differences in the prevalence of OCD among the participants in the present study were considerable because of the different hospitals. As mentioned earlier, hospitals A, B, and C were located in three different regions in the north, center, and south of Tehran, respectively, with significant socioeconomic differences. That is, the socioeconomic status of residents, and thus their access to health services, worsens when they are transferred from the northern region to the southern region of Tehran. Accordingly, the prevalence of OCD among individuals treated in hospitals A and C (in the northern and southern regions of Tehran, respectively) (72.3% overall and 36.15% individually) was higher than in hospital B (27.69%). Also, in the 2021 study by Garnier et al., socioeconomic inequalities in the form of more individuals below the poverty line were associated with a higher risk of individuals with COVID − 19 in the United States [35]. However, the higher prevalence of OCD among individuals treated in hospital A (36.15%) compared with hospital B (27.69%) was substantial, which was inconsistent with the explained differences in accessibility to health services due to regional disparities. Although a congruent study was conducted by Kahneman et al. 2021 that emphasized the helplessness of higher socioeconomic status in improving emotional well-being [36].

Regarding “gender”, the prevalence of OCD was generally significantly higher in women (127 subjects) than in men (86 subjects). Even when considering the frequency of each gender in the study separately, the prevalence of OCD was still higher in females (76.5% of the total frequency in females) than in males (64.17% of the total frequency in males). This is consistent with the findings of the study by Abba-Aji et al. that women are significantly more likely to have OCD compared to men [33]. The review article (2019) showed that gender may play a role in the onset, presentation, and impact of OCD symptoms. Overall, it is thought to be more common in females during adolescence and adulthood. In men, symptoms tend to occur earlier and are associated with blasphemous thoughts. Females often describe symptom onset during or after puberty or pregnancy and present with symptoms related to contamination and/or aggressive obsessions [37].

In addition, the prevalence of OCD was highest in individuals in whom 6 to 12 months had elapsed since the onset of COVID 19 compared with the other time-related groups, but was not significant.

Regarding “marital status,“ OCD was generally more common in married individuals (188 individuals) than in single individuals (25 individuals). It should be noted, however, that the overall frequency of married persons participating in the present study was impressively high. Nevertheless, the prevalence of OCD was still slightly higher among married persons (71.21% of the total frequency of 264 married persons) than among single persons (69.44% of the total frequency of 36 single persons) when frequency by marital status was considered separately. Accordingly, as claimed by Abba-Aji, married individuals had a higher prevalence of OCD [33].

Notwithstanding the fact that most of the participants in the present study had a higher level of education (university) (179 subjects), subjects with primary level of education and then with secondary level of education (high school) had the highest prevalence of obsessive-compulsive disorder (78.78% of 66 subjects and 76.36% of 55 subjects, respectively), considering the frequency of each educational group. Accordingly, the prevalence of obsessive-compulsive disorder was lowest in individuals with higher educational level (66.48% out of 179 individuals), although the difference was not significant. In this regard, the study of Abba-Aji showed that the prevalence of OCD was higher in individuals with higher educational level than in individuals with primary and secondary educational level [33]. Moreover, as claimed by Liao et al. (2021), the prevalence of OCD was higher in individuals with higher educational level one year after recovery COVID − 19. Although the mentioned difference was not meaningful [38].

Since most of the participants in the current study were born in Tehran (288 individuals), it is normal that they had the highest prevalence of OCD, regardless of the significance of the reported difference. Therefore, the reported result was questionable.

Finally, individuals with an ICU history (85.29% of 34 individuals) and intubation (87.5% of 24 individuals) during COVID 19 also had a higher prevalence of OCD after recovery from COVID 19 than individuals without the aforementioned records. However, none of the reported differences in ICU history and intubation were significant. Interestingly, despite the fact that most of the participants in the present study had undergone hospitalization (238 individuals), the prevalence of OCD was higher in those who had not undergone hospitalization (74.19% of 62 individuals). However, the observed difference was not significant. There is no question that certain conditions such as past hospitalizations, ICU admission, and aggressive procedures such as intubation may be associated with significant psychological tension. Therefore, a higher prevalence of mental disorders such as obsessive-compulsive disorder was expected in the aforementioned individuals. Accordingly, the results of a review study conducted by Pinto et al. indicated that surgical complications, as an invasive procedure, have a significant impact on the psychosocial well-being of the affected individuals [39]. Furthermore, as Rose et al. assert, ICU admission has been associated with significant short- and long-term psychological distress, including delirium, anxiety, depression, and PTSD [40]. In contrast to the data in the present study on hospitalization, Bellan et al. stated that individuals hospitalized for COVID were more likely to have striking psychological sequelae [41].

Finally, a significant association was found between OCD and stress, OCD and depression, OCD and anxiety, OCD and PTSD, and finally OCD and sleep disorders. In other words, the above factors may strongly predict OCD after COVID infection. Accordingly, the results of a cross-sectional study conducted by Atreya et al. in 2020 indicated the correlation between “OCD and stress”, “OCD and anxiety”, and finally “OCD and cognitive dysfunction” [42]. Moreover, the results of the study conducted by Sirvastava et al. 2022 indicated the existence of a correlation between “OCD and anxiety” and “OCD and stress” [43]. It appears that psychological distress such as stress, anxiety, depression, and PTSD may increase the risk of OCD. It is known that stress with disturbances in the corticostriatal and limbic circuits can lead to neural abnormalities that result in imbalances in goal-directed and habitual behaviours [44].

## Strengths and limitations

Our study was a follow-up study to contribute to a more comprehensive assessment of COVID − 19 recovered individuals to identify and appropriately treat their psychological area. It may help to expand structured community-based mental health interventions in post-COVID era. As with any investigation, the present study had some limitations. The small sample size of the study in each urban region is one of the limitations. Another limitation of the study is response bias. out of 1103 potential participants, only 339 people answered the entire questionnaire. The mentioned limitation in the form of voluntary participation in the study due to the restrictions of the ethical code limited the generalizability of the data from our COVID − 19 recovered population. The lack of information about the family history of OCD or other psychiatric disorders in patients may also affect the clarity of COVID-related obsessive-compulsive disorder results. In conclusion, it is recommended that further longitudinal studies be conducted to examine the course of COVID-centered OCD and to assess the impact of evidence-based interventions on key predictors. The study did not provide information on duration of OCD symptoms, treatment, or follow-up, so future studies in this area may be informative. There is also a need for more studies on older adults and other age groups.

## Conclusion

​Because the OCI-R is a self-assessment instrument, it should be noted that nearly 70% of COVID − 19 recovered individuals were found to have OCD-like symptoms of mild to moderate severity and were not necessarily diagnosed with obsessive-compulsive disorder. In addition, the prevalence of OCD was higher in subjects who were older, female, married, unemployed, residing in the capital city, with a lower SaO2, 6 to 12 months after onset, not hospitalized, hospitalized in regions of low and high socioeconomic status, with more hospital days, in the intensive care unit, and with a history of intubation, although the importance of the above aspects varied. Finally, there was a significant correlation and predictive power between OCD and stress, anxiety, depression, sleep disorder, and PTSD.

## Data Availability

The data sets used during the current study can be provided by the corresponding author [F.G], upon reasonable request.
